# Adjunctive Hyperbaric Oxygen Therapy During Femoral Limb Lengthening in a Child: A Case Report

**DOI:** 10.7759/cureus.117129

**Published:** 2026-09-29

**Authors:** Anirejuoritse Bafor, Bicheng Yong, Christopher Iobst

**Affiliations:** 1 Orthopedic Surgery, Nationwide Children's Hospital, Columbus, USA

**Keywords:** bone healing, distraction osteogenesis, hyperbaric oxygen therapy, limb lengthening, limb reconstruction surgery

## Abstract

Hyperbaric oxygen therapy (HBOT) has several established clinical applications, including treatment of severe anemia, crush injuries, and necrotizing soft tissue infections. Increasing interest also exists in its potential to enhance bone regeneration, as demonstrated in animal models. Improved regenerate formation and maturation could shorten treatment time during distraction osteogenesis (DO). We describe the feasibility and application of HBOT as an adjunct during femoral limb lengthening with a magnetic lengthening nail in a pediatric patient.

A pediatric patient underwent two sequential femoral lengthening procedures with a magnetic lengthening nail at ages 6 and 10 years. HBOT was used as a patient-initiated, self-referred adjunctive therapy during both treatments to promote regenerate bone formation. Outcomes assessed included pixel value ratio (PVR), bone healing index (BHI), time to full weight-bearing (FWB), lengthening rate and duration, radiographic and clinical findings, hardware function, and complications. The patient completed 46 HBOT sessions during the first lengthening and 36 during the second. Regenerate PVR remained consistently above 0.85 relative to surrounding bone throughout distraction and consolidation. The length gained was 4.0 cm and 4.8 cm, respectively. BHI was 27.25 days/cm for the first procedure and 18.54 days/cm for the second. Full weight-bearing was achieved by 12 weeks postoperatively.

This case demonstrates the novel use of HBOT as an adjunct in human lower extremity DO. In this patient, HBOT was feasible and associated with robust regenerate formation and early recovery. Further study is warranted to determine whether HBOT can improve outcomes in limb lengthening patients.

## Introduction

Distraction osteogenesis (DO) is widely used to treat leg length discrepancy. It involves an osteotomy followed by a gradual separation of two vascularized bone segments, allowing new bone to form and mineralize within the resulting gap [1-3]. A key limitation is the prolonged, variable consolidation phase, which can delay full weight-bearing and increase the risk of stiffness, deformity, and fracture [4]. Biologic or mechanical adjuncts that enhance regenerate maturation may shorten treatment and reduce these complications.

Hyperbaric oxygen therapy (HBOT) involves intermittent breathing of 100% oxygen at pressures >1 atmosphere absolute and has been used as an adjunct in orthopedics for musculoskeletal infection, impaired wound healing, nonunion, compromised flaps, and other complex conditions [5,6]. Animal studies suggest that HBOT can increase bone mineral density during DO and mitigate adverse effects on skeletal muscle [7,8]. However, its use during internal limb lengthening in children has not been described.

We describe a child who underwent two sequential femoral lengthening procedures using a motorized nail, first off-label, extramedullary, and later, intramedullary. Adjunctive HBOT was administered during each treatment course based on self-referral by the caregivers of the child. The aim of this report is not to demonstrate the efficacy of HBOT as a treatment adjunct to DO, but to detail the clinical and radiographic course, demonstrate the feasibility and tolerance of HBOT in this complex post-traumatic setting, and provide hypothesis-generating observations to inform potential future studies of HBOT in limb lengthening.

## Case presentation

A six-year-old boy sustained a Gustilo type III-B open distal femoral fracture [9] with transphyseal separation, partial loss of the lateral femoral condyle, and a large soft tissue defect from a lawnmower injury, initially treated with irrigation/debridement, serial vacuum-assisted closure (VAC) dressings, and distal thigh skin grafting, three years before presenting at our facility.

He presented to us with a 3-cm limb length discrepancy (right longer than left) and left knee valgus (lateral distal femur angle (LDFA): 75°) (Figure 1). Given the small femoral canal and the family’s wish to avoid external fixation, a motorized lengthening nail was used off-label as an extramedullary device after detailed counseling and shared decision-making, using a previously described technique [10].

**Figure 1:**
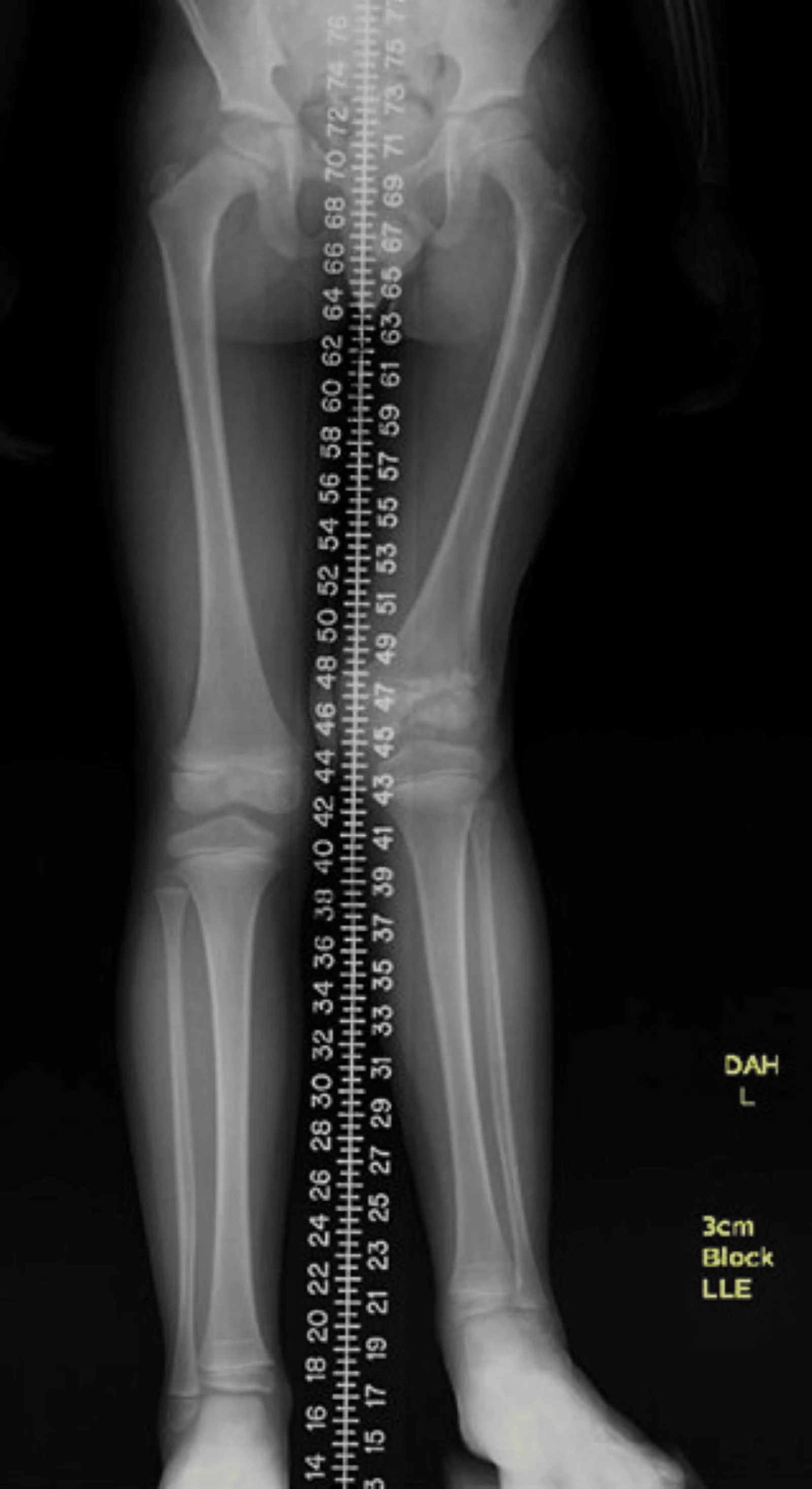
Full-length standing anteroposterior radiograph obtained 3 years after a lawnmower injury to the left lower extremity, demonstrating a 3-cm limb length discrepancy (right longer than left) with a 3-cm block under the left foot and valgus deformity of the left knee

First lengthening (extramedullary nail)

After a 7-day latency period, distraction began at 0.75 mm/day (0.1875 mm in 4 increments), was increased to 1.0 mm/day for 2 weeks, and briefly to 1.2 mm/day in response to robust regenerate formation. Discomfort prompted a return to 1.0 mm/day (0.25 mm in 4 increments) until 4.0 cm of femoral lengthening was achieved.

HBOT was not prescribed as part of our standard limb lengthening protocol. Rather, the patient/family independently pursued HBOT through self-referral as an adjunctive measure during treatment. HBOT was delivered in a Class B monoplace chamber (Sechrist 3300H; Sechrist Industries, Inc., Anaheim, CA) (Figure 2) at 2.0 ATA for about 75 minutes per session, under technologist supervision and with post-treatment monitoring.

**Figure 2:**
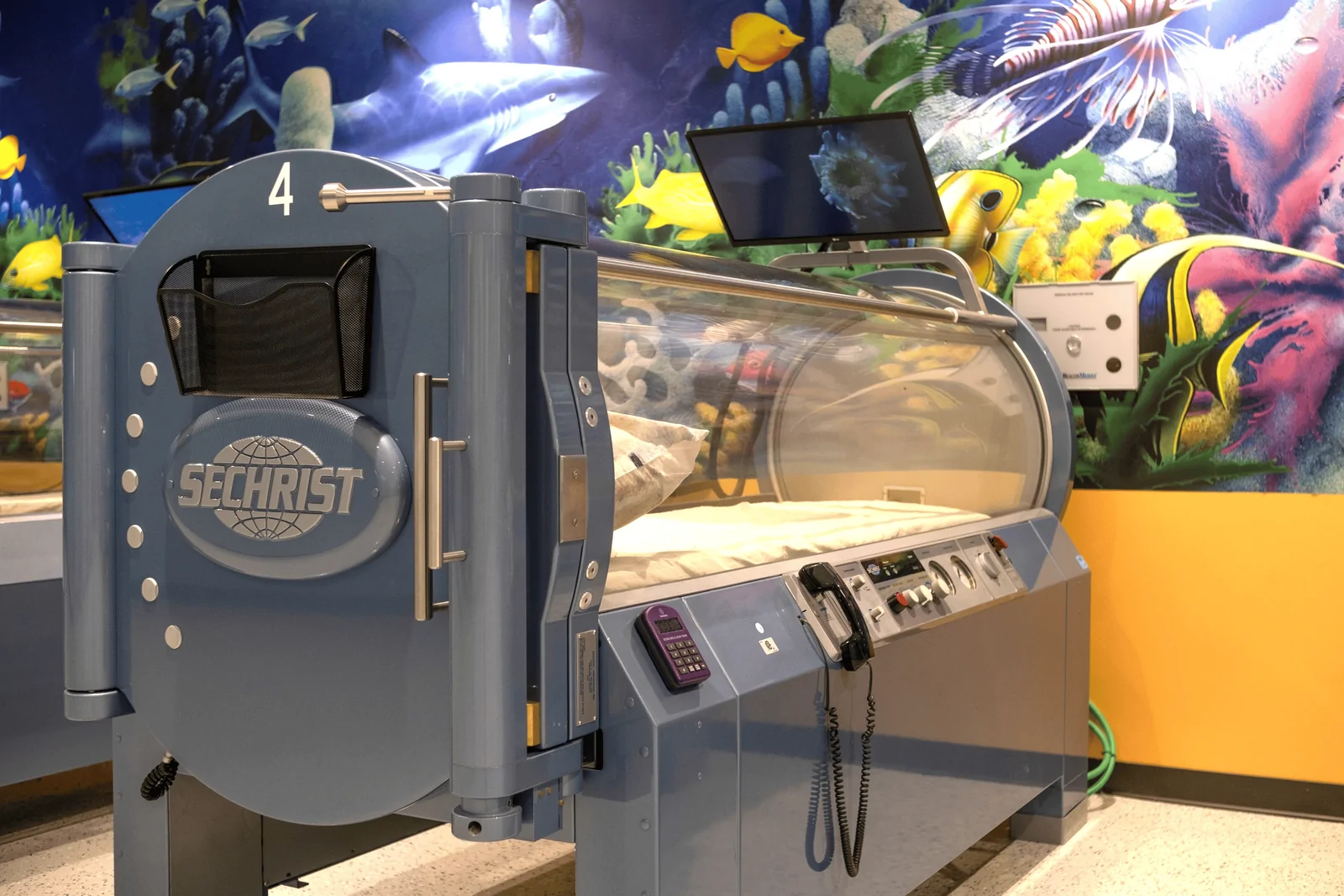
Pediatric Class B monoplace hyperbaric oxygen chamber (Sechrist 3300H; Sechrist Industries, Inc., Anaheim, CA) used for adjunctive hyperbaric oxygen therapy in this case Photograph courtesy of The Oxford Center, Brighton, Michigan, USA; reproduced with permission

The patient completed 45 sessions (2 preoperative, 35 during distraction/consolidation, and 8 after nail removal) (Figure 3); pixel value ratio (PVR) improved from 0.85 at 2 weeks to 0.97 by 8.5 weeks, full weight-bearing was allowed at 15.5 weeks, the nail was removed at 21 weeks (Figure 4a-4o), and the bone healing index (BHI) was 27.25 days/cm.

**Figure 3:**
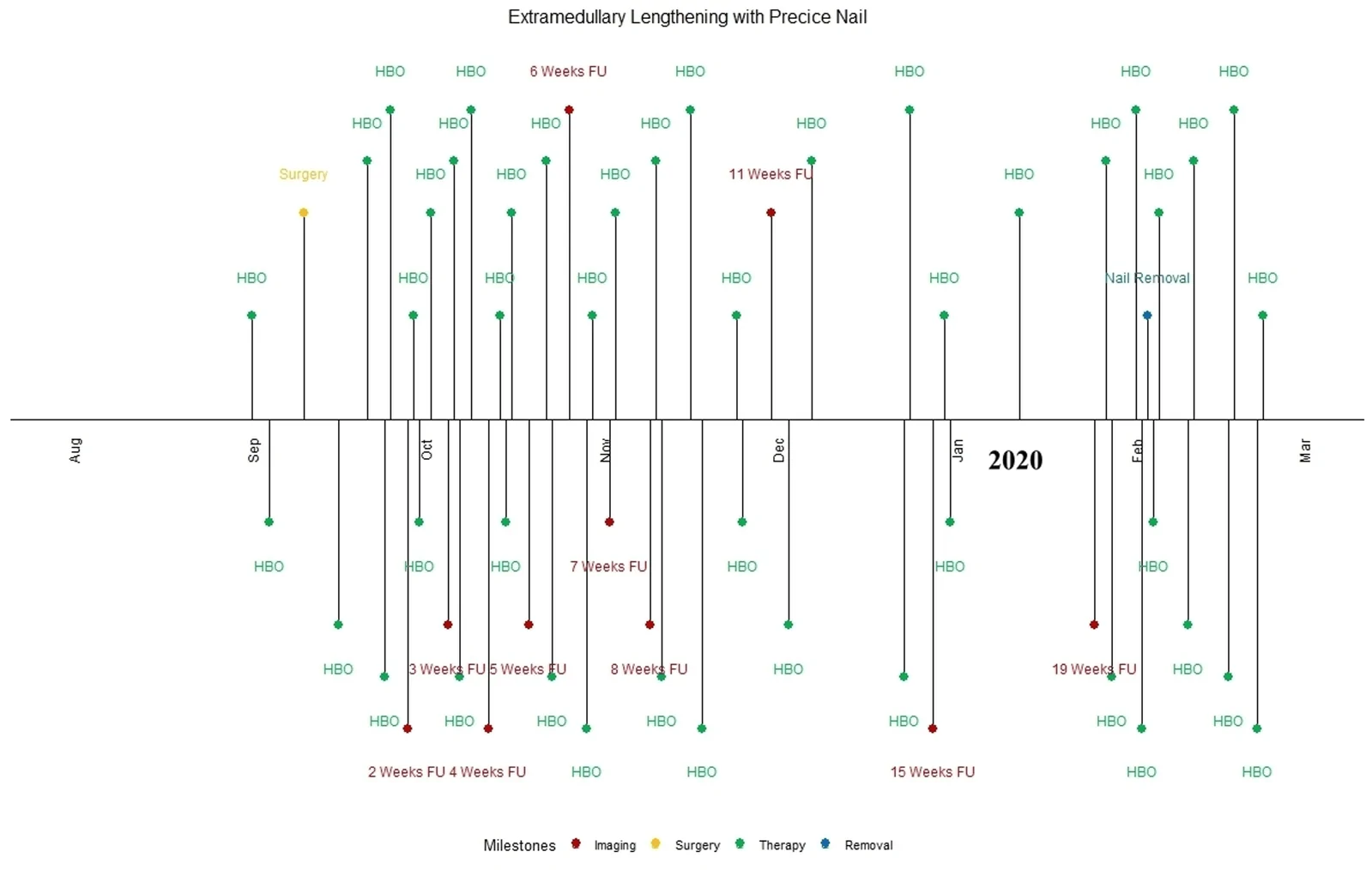
Timeline of HBOT sessions and key clinical milestones (surgery, follow-up visits, and nail removal) during the first extramedullary femoral lengthening with a magnetic PRECICE nail The figure was generated using R statistical software (R Foundation for Statistical Computing, Vienna, Austria) and the ggplot2 package. HBOT: hyperbaric oxygen therapy

**Figure 4:**
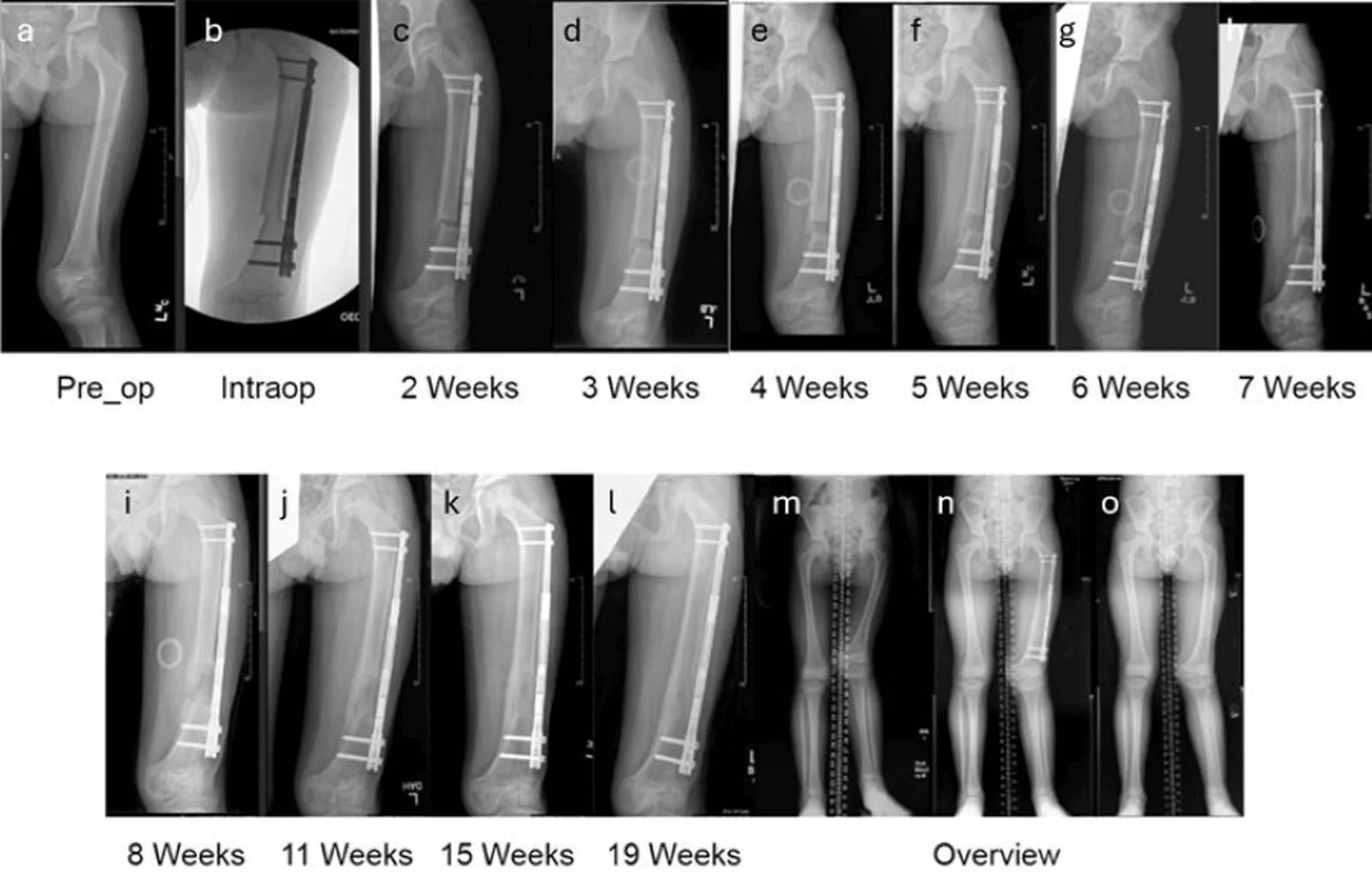
Sequential anteroposterior radiographs demonstrating extramedullary femoral lengthening with a retrograde magnetic nail (a) Preoperative; (b) intraoperative; (c-l) 2-, 3-, 4-, 5-, 6-, 7-, 8-, 11-, 15-, and 19-week postoperative radiographs, respectively, showing progressive regenerate formation and consolidation; (m-o) standing lower extremity overview radiographs demonstrating overall alignment

Second lengthening (intramedullary nail)

By age 10, standing radiographs showed recurrence of a 4-cm limb-length discrepancy (right longer than left) and genu valgum (LDFA: 80°) (Figure 5). The femoral canal was now large enough for the smallest intramedullary lengthening nail. A retrograde intramedullary magnetic nail was therefore planned using a modified reverse planning method to correct both deformity and discrepancy [11].

**Figure 5:**
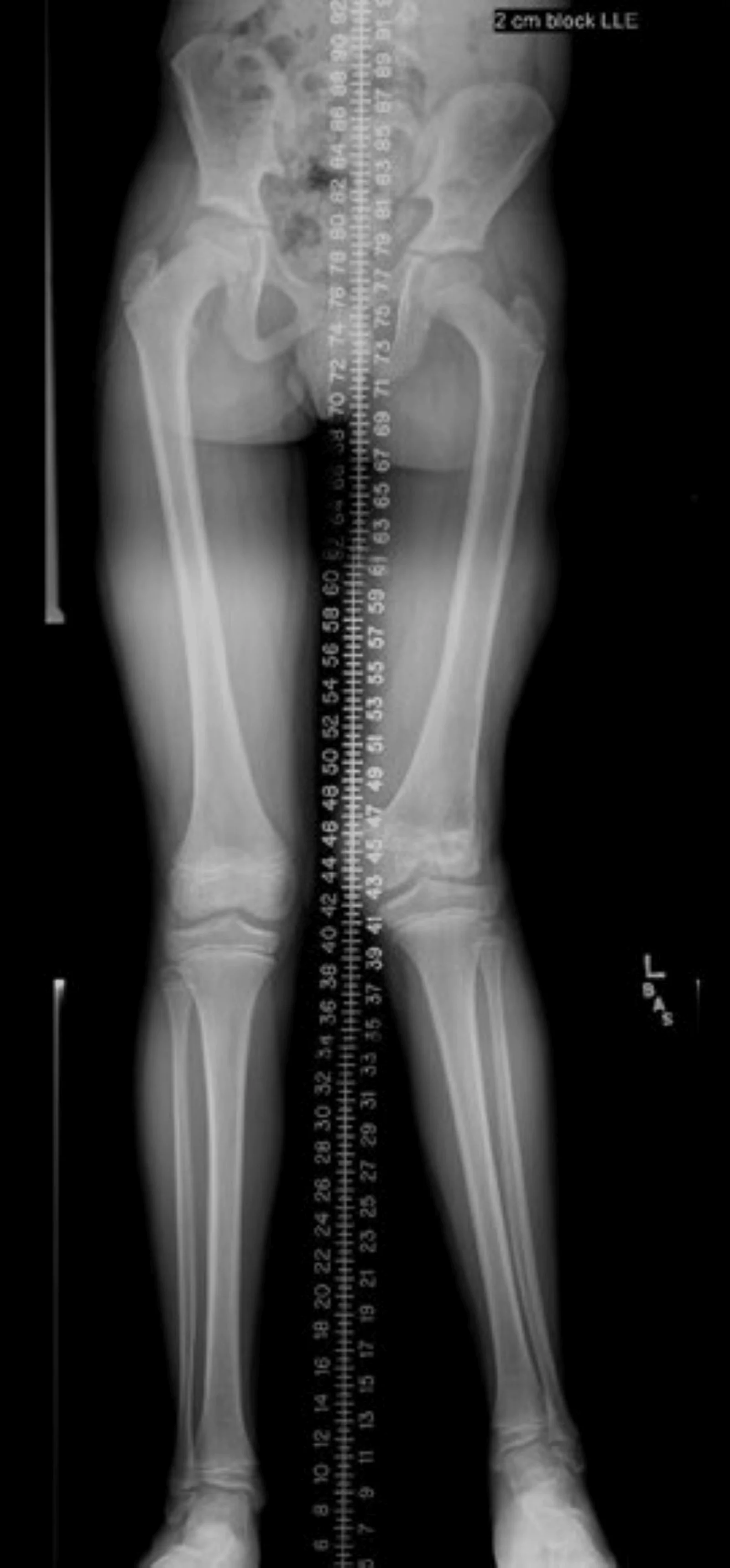
Full-length standing anteroposterior radiograph at age 10 years, with a 2-cm block under the left foot, demonstrating recurrence of a 4-cm limb-length discrepancy (right longer than left) and persistent valgus deformity of the left femur

After a 7-day latency, distraction started at 0.8 mm/day and was increased to 0.9 mm/day in the final week, achieving 4.8 cm of lengthening in about 7 weeks. He received 35 HBOT sessions (4 preoperative, 22 during distraction/consolidation, and 9 after nail removal) (Figure 6); PVR improved from 0.82 at the end of distraction to 1.0 by 12 weeks, full weight-bearing was allowed at that time, the nail was removed at 35 weeks (Figure 7a-7l), the BHI was 18.54 days/cm, and knee motion remained full without contracture or extensor lag.

**Figure 6:**
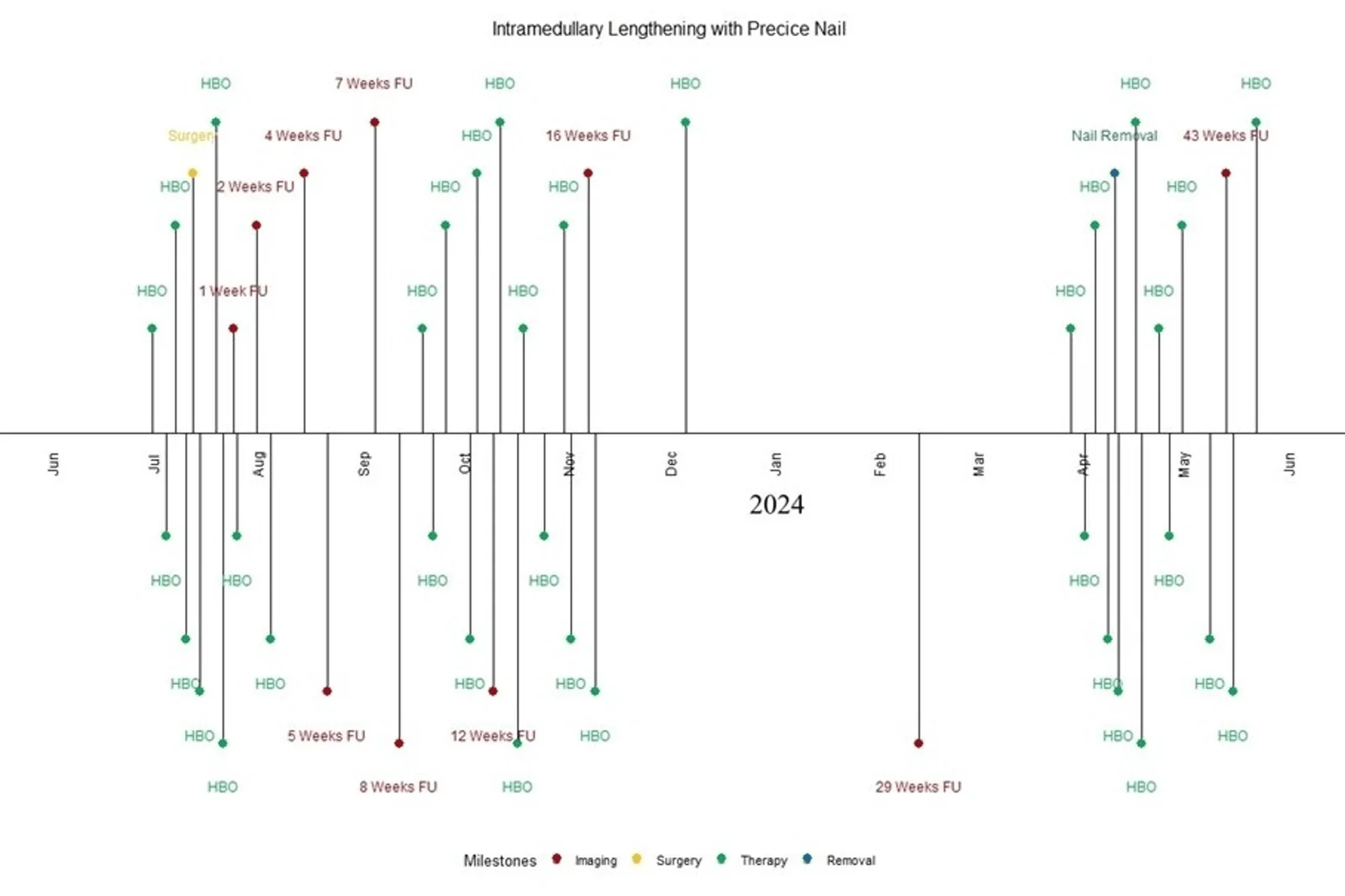
Timeline of HBOT sessions and key clinical milestones (surgery, follow-up visits, and nail removal) during the second intramedullary femoral lengthening with a magnetic PRECICE nail The figure was generated using R statistical software (R Foundation for Statistical Computing, Vienna, Austria) and the ggplot2 package. HBOT: hyperbaric oxygen therapy

**Figure 7:**
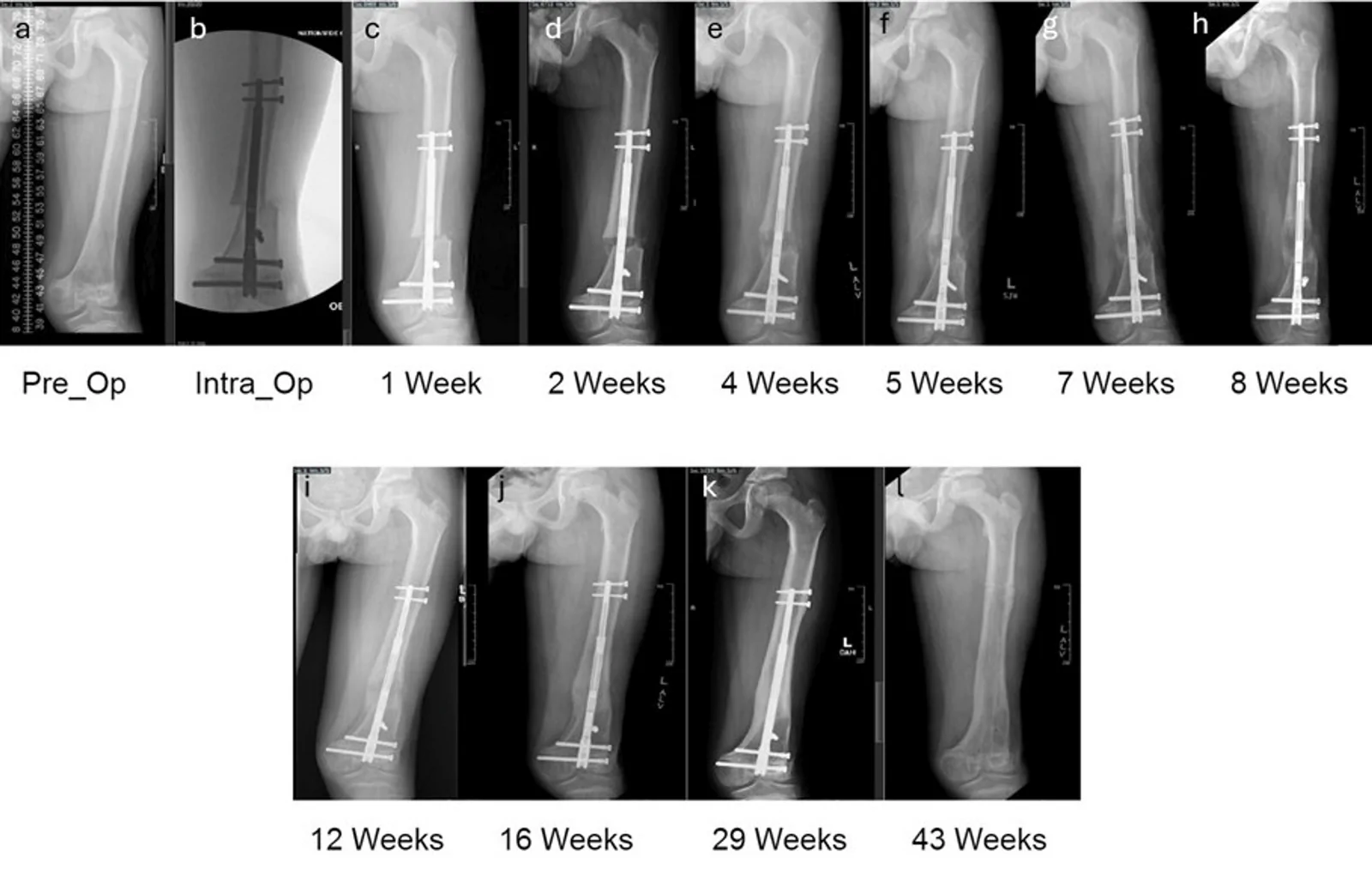
Sequential anteroposterior radiographs demonstrating acute correction of distal femoral valgus deformity followed by retrograde intramedullary lengthening with a magnetic nail using the modified reverse planning method (a) Preoperative; (b) intraoperative; (c-k) 1-, 2-, 4-, 5-, 7-, 8-, 12-, 16-, and 29-week postoperative radiographs, respectively, showing progressive regenerate formation and consolidation; (l) 43-week radiograph after nail removal following satisfactory consolidation

Monitoring and quantitative assessment

The patient had weekly clinical and radiographic reviews during distraction and 3-4-weekly visits during consolidation, with distraction rates adjusted to regenerate quality. A night knee extension brace, early touch-down weight-bearing, and focused physiotherapy were used, with weight-bearing advanced once cortical continuity was seen radiographically. BHI (days/cm) and PVR were calculated from digital radiographs.

Patient consent

Written consent for publication of this case report and images was obtained from the patient’s parents.

## Discussion

We report a child with post-traumatic partial distal femoral growth arrest, genu valgum, and progressive limb length discrepancy who underwent sequential extramedullary and intramedullary femoral lengthening with adjunctive HBOT. Importantly, HBOT in this case was patient-initiated rather than incorporated into our routine treatment algorithm or prescribed in response to a specific clinical indication. This should be considered when interpreting the observed course, as the report is descriptive and does not support any conclusion regarding the indicated or routine use of HBOT during distraction osteogenesis. To our knowledge, this is the first clinical description of HBOT used with an internal lengthening nail in a pediatric patient.

HBOT substantially increases dissolved oxygen and tissue oxygen tension [5,6,12]. Experimental work has demonstrated enhanced collagen synthesis, angiogenesis, osteoblast activity, and more robust regenerate formation under hyperbaric conditions, with animal DO and defect models showing increased bone mineral density and reduced fibrous tissue [7,8,13-17]. These data provide a biologic rationale for using HBOT to support regenerate formation in DO.

In our patient, regenerate maturation appeared relatively rapid, with PVR ≥ 0.82 by the end of distraction for both procedures and approaching or reaching 1.0 by 8.5-12 weeks, without fracture, nonunion, or stiffness. Elsheikh et al. reported that, after PRECICE femoral lengthening, full weight-bearing was typically allowed a median of 42 days after distraction once PVR reached approximately 0.85, without increased implant failure or regenerate fracture [18]. In this case, full weight-bearing was permitted at 12-15.5 weeks, coinciding with high PVR values and uneventful consolidation. We, however, cannot infer causality from a single case and agree that the observation of favorable PVR values in this report warrants further investigation.

The BHIs in this patient were also positively enhanced compared with published reports. Hafez et al. reported mean femoral BHI values of 53.2±19 days/cm and 40.2±14 days/cm for external fixator and intramedullary lengthening, respectively [19]. Higher indices are associated with functional or anatomic complications [20]. In contrast, BHIs of 27.25 and 18.54 days/cm were observed in our patient, together with preservation of knee motion and absence of regenerate-related complications. However, these observations are descriptive only and should not be interpreted as evidence of superiority over previously reported outcomes. Given the single-case observational design, no causal inference can be made regarding the contribution of HBOT. The favorable course likely reflects the influence of multiple factors, including young age, absence of significant comorbidities, meticulous planning, and structured rehabilitation. Accordingly, any role of HBOT in this setting remains hypothesis-generating.

The HBOT regimen evolved between procedures, with 45 sessions during the first lengthening and 35 during the second, delivered before surgery, during treatment, and after nail removal. This adaptive approach reflected real-time assessment of progress rather than a fixed protocol. It is also possible that the initial, more intensive course of HBOT improved the local tissue environment before the second lengthening. However, interpretation is confounded by differences in technique (extramedullary versus intramedullary) and patient age.

The balance between bone and soft tissue adaptation is crucial in DO. HBOT may augment regenerate formation, but musculotendinous and neurovascular structures must also accommodate progressive lengthening. During the first lengthening, acceleration of the distraction rate in response to rapid regenerate formation led to discomfort and prompted a rate reduction. This underscores the importance of conservative adjustments to the distraction rate and careful clinical monitoring when using HBOT, so that enthusiasm for bone healing does not outpace soft tissue tolerance.

Despite promising biologic rationale, the role of HBOT in bone healing remains uncertain. Some experimental and clinical studies have not demonstrated clear benefits for fracture healing or nonunion management, and systematic reviews have not identified high-quality evidence to support or refute its use in delayed or nonunion fractures [12,16,21,22]. Optimal timing, duration, and frequency of HBOT in relation to osteotomy and distraction also remain undefined [7,23-25]. Most data derive from animal models, and translation to pediatric DO is not straightforward. Practical barriers further limit widespread adoption. HBOT requires specialized facilities, trained staff, and repeated 60-90-minute sessions over weeks and can be costly. Insurance coverage is typically restricted to a limited set of approved indications, and adjunctive use during limb lengthening may not be reimbursed. These logistical and economic constraints likely contribute to the rarity of HBOT in limb lengthening protocols, even when biologically plausible.

This case report has several limitations. Because this is a single-case report without an internal control, the contribution of HBOT to the observed bone regeneration cannot be isolated from other factors, including the patient’s young age, surgical technique, stable fixation, and inherent healing potential, which introduce additional confounding. We also cannot quantify the incremental contribution of HBOT relative to careful surgical execution, distraction control, and rehabilitation. Therefore, the rapid maturation of the regenerate observed in this case should not be interpreted as evidence of efficacy. Rather, this report demonstrates that HBOT may be safely incorporated into distraction osteogenesis and provides preliminary clinical observations that support future prospective studies evaluating its potential role in enhancing regenerate bone formation. An additional limitation of this case report is that pain was not assessed as a predefined outcome measure. No standardized pain scores, patient-reported pain assessments, or analgesic consumption data were collected during the distraction or consolidation phases. As a result, we are unable to determine whether adjunctive HBOT had any effect on pain experience, pain control, or analgesic requirements during the lengthening process. This is relevant because pain is an important clinical outcome in limb lengthening and may influence patient tolerance, rehabilitation, and overall treatment burden. Accordingly, any potential effect of HBOT on pain in this setting remains unknown and should not be inferred from the present report. Future studies evaluating HBOT during distraction osteogenesis should prospectively include standardized pain-related outcome measures in order to better define its potential symptomatic and biologic effects.

Larger, prospective studies will be required to determine whether the favorable healing observed here is reproducible and clinically meaningful in broader populations.

## Conclusions

In this pediatric patient with post-traumatic distal femoral growth arrest treated with sequential femoral lengthening, adjunctive HBOT was feasible and well tolerated. Although satisfactory regenerate formation and recovery were observed, the single-case observational nature of this report does not permit attribution of these findings to HBOT. HBOT was not part of our routine limb lengthening protocol, and this case should not be interpreted as supporting its routine or indicated use in distraction osteogenesis. Instead, it serves as a descriptive, hypothesis-generating observation that may inform future prospective investigation.
